# Investigation of effects of different colour led lighting application to pregnant rats on mother and offspring

**DOI:** 10.1590/1980-220X-REEUSP-2024-0398en

**Published:** 2025-07-07

**Authors:** Halime Aydemir, Fatma Ergün

**Affiliations:** 1 Kirsehir Ahi Evran University, Faculty of Health Sciences, Department of Midwifery, Kirsehir, Türkiye.; 2 Kirsehir Ahi Evran University, Faculty of Health Sciences, Department of Nutrition and Dietetics, Kirsehir, Türkiye.

**Keywords:** Rat, Light, Pregnancy, Red Light, Offspring, Rato, Luz, Gravidez, Luz Vermelha, Filhotes

## Abstract

**Objective:**

In the study, the effects of different colour LED lighting on the physiological and behavioural characteristics of pregnant rats, mother rats, and their offspring were investigated using an experimental model consisting of primiparous (first-time delivering) rats.

**Method:**

In the study, 24 pregnant rats (Wistar Albino) were included in 4 groups, each including. The rats were placed in 4 light cabinets (White, Red, Blue, Yellow). During pregnancy and the postpartum period, the body weights of the pregnant/mother rats and their offspring, the weight gain of the pregnant rats, the daily feed and water consumption, blood glucose levels, skin thicknesses, and the behavioural characteristics of the pregnant rats and their offspring were assessed.

**Results:**

Feed and water consumption was highest in the Red group (0.51 ± 0.06 ml/BW (g)/day). Significant differences were determined between the rat groups in terms of their blood glucose levels, skin thicknesses, and the results of the behavioural characteristics on the pregnant and offspring rats (p < 0.05).

**Conclusion:**

Red light had a weight-increasing effect on pregnant rats, while blue light had an oedema-preventing effect and a weight-increasing effect on the offspring.

## RESUMO

**Objetivo:**

No estudo, foram investigados os efeitos de diferentes cores de iluminação LED sobre as características fisiológicas e comportamentais de ratas prenhes, ratas-mães e seus filhotes, usando um modelo experimental composto por ratas primíparas (que deram à luz pela primeira vez).

**Método:**

No estudo, 24 ratas prenhes (Wistar Albino) foram incluídas em 4 grupos, cada um deles com um animal. As ratas foram colocadas em 4 gabinetes de luz (branco, vermelho, azul e amarelo). Durante a gravidez e o período pós-parto, foram avaliados os pesos corporais das ratas grávidas/mães e de seus filhotes, o ganho de peso das ratas grávidas, o consumo diário de ração e água, os níveis de glicose no sangue, a espessura da pele e as características comportamentais das ratas grávidas e de seus filhotes.

**Resultados:**

O consumo de ração e água foi maior no grupo vermelho (0,51±0,06 ml/BW (g)/dia). Foram determinadas diferenças significativas entre os grupos de ratos em termos de seus níveis de glicose no sangue, espessura da pele e os resultados das características comportamentais das ratas prenhes e dos filhotes (p&lt;0,05).

**Conclusão:**

A luz vermelha teve um efeito de aumento de peso em ratas prenhes, enquanto a luz azul teve um efeito de prevenção de edema e um efeito de aumento de peso na prole.

## INTRODUCTION

Improving maternal and newborn health and reducing maternal and neonatal morbidity and mortality are of great importance for the health of pregnant women^(1)^. Pregnancy is considered as a critical period for maternal and newborn health.

Providing care based on evidence-based guidelines improves the quality of care^(2)^. The World Health Organisation (WHO) recommendations for positive pregnancy experience in antenatal care include healthy nutrition and exercise, daily iron and folic acid supplementation, daily calcium supplementation, and limiting caffeine intake above 300 mg per day. Recommendations for pregnant women include anaemia diagnosis, early ultrasound, glucose tolerance test, screening for tobacco use and passive smoking, partner violence, asymptomatic bacteriuria with 7 days of antibiotics, anti-D immunoglobulin, and preventive anti-helminth treatment. The WHO also recommends that every pregnant woman should have her own health information, tetanus vaccination, the number of antenatal visits, and recommendations to prevent common pregnancy complaints (hyperemesis gravidarum, heartburn, cramps, back and pelvic pain, constipation, varicose veins and oedema)^(3)^. These recommendations are aimed ensuring a healthy progression of pregnancy. However, even with a healthy pregnancy monitored, there are inevitable factors in daily life, one of which is light.

Light is an indispensable source for sustaining our lives as long as we are awake^(4)^. During pregnancy, a woman are faced with the effects of lighting systems on pregnancy, childbirth, and postpartum processes. Although the LED lights used in lighting today are considered in monitoring and care in line with WHO recommendations, they are a factor that can affect the pregnancy process. Given the necessities of human life, it is a reality that it is difficult to eliminate the effects of lighting under current conditions.

It has been reported that blue LED lights affect bilirubin levels and shorten the treatment time in phototherapy treatment^(5)^. Phototherapy has been used for many years as a reliable and effective method in the treatment of neonatal jaundice^(6)^. This experimental study was designed to monitor and assess the effects of LED lights on rats. The study is expected to shed light on studies on how LED lights affect other mammals such as rats.

## METHOD

In the study, 24 primiparous (first-time pregnant) female rats (Wistar Albino) aged 14–15 weeks (Body Weight (BW) 200 ± 15 g), with similar morphological characteristics, raised in the Experimental Animals Unit of the Faculty of Medicine, Kirsehir Ahi Evran University, were used.

**Figure 1:**
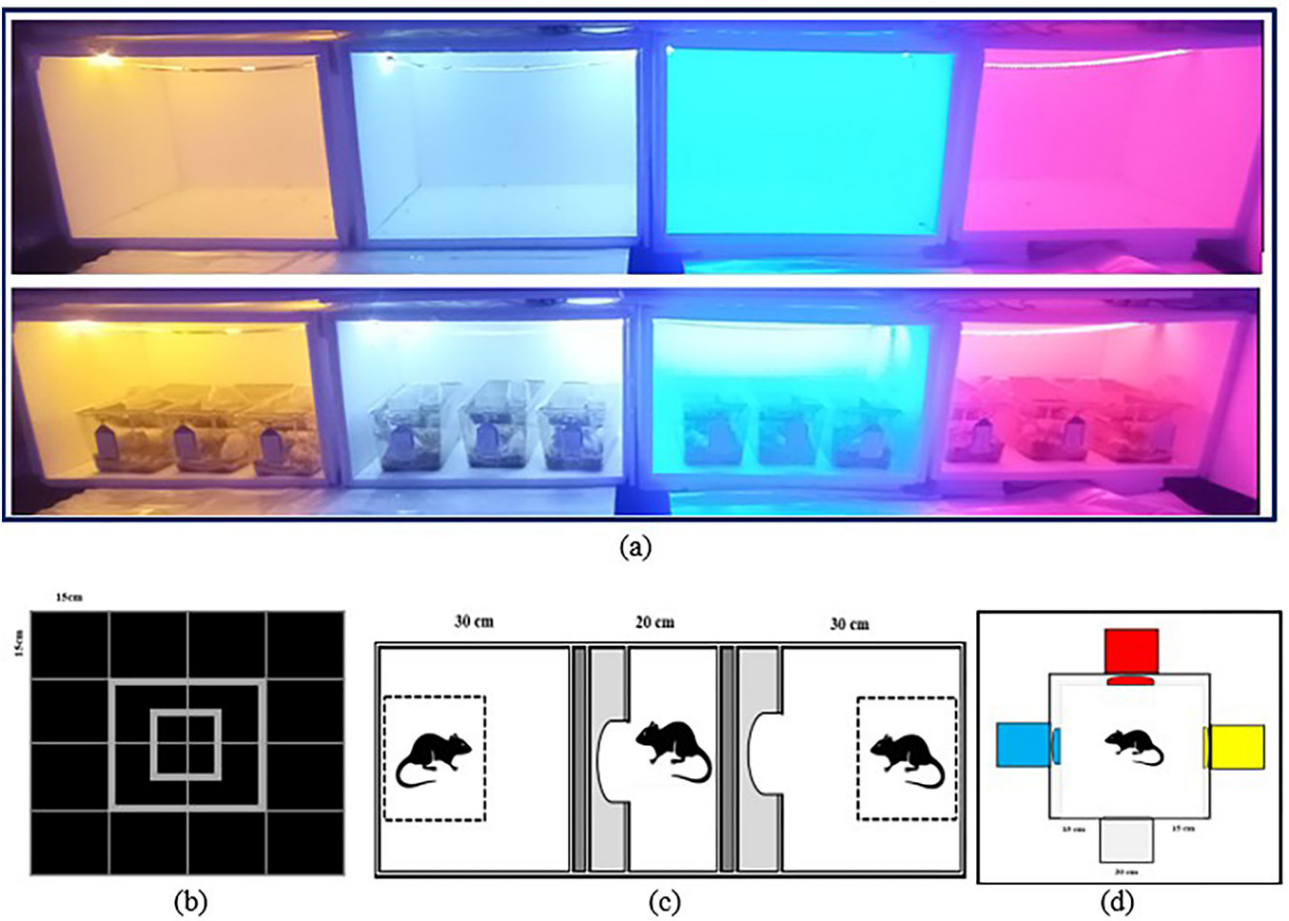
Cabinets illuminated with different coloured LED lights and behavioural analysis test platforms (a; Breeding cages, b; Open field test platform, c; Social preference test platform, d; Light reflex test platform).

The entire stages of the study were carried out in an environment with a temperature of 22 ± 2 °C, a relative humidity of 35 ± 10%, and a noise level of 35 ± 10 decibels (dB)(7). The study environment was illuminated with white light in a 12-hour light/12-hour dark cycle. For the female rats to become pregnant, 24 female rats were placed in pairs in 12 mating cages. One 18–20-week-old male rat was placed in each cage (2 females/1 male). After the male rats were kept in the cages for 6 days (the estrous cycle in rats is 4–5 days), they were removed from the cages^(8)^. The female rats were then divided into 4 groups, each with 6 rats in a homogeneous manner. The 6 rats in each group were placed in 3 breeding cages (425*265*150/800 cm^2^)^(9)^ which were placed in 4 specially prepared light cabinets (White, Red, Blue, Yellow) for the study. The cabinets were made of white Expanded Polystyrene (TS7316- EN13163) with dimensions of 100 × 50 × 50 cm, with the front parts open and light-impermeable. LED light sources were installed on the ceiling of the cabinets to provide a light intensity of 150 ± 25 lux on the floor, in addition to the 12-hour light/12-hour dark cycle, the cabinets were illuminated with white (400–740 nm), red (635–700 nm), blue (450–490 nm), and yellow (560–590 nm) lights for 4 hours^(10)^. The additional light application was carried out between 7:00 p.m. and 11:00 p.m. following the light period (Figure 1-a).

The light intensity and duration periods utilized were inspired by typical home and office lighting patterns (illuminated environment from dusk until midnight). Furthermore, the continuous white lighting commonly used in domestic and office settings was designated as the control group in the study. The light intensity within the cabinets was monitored every 3 days using a digital lux meter (Extech 5-in-1 Environmental Meter EN300).

During the gestational and postpartum periods, the BW’s of the pregnant/mother rats and their offspring were measured using a digital scale (NECK WT-NF Precision Scale) accurate to 0.01 units, and the daily BW gains (g/day) were calculated. Additionally, the daily feed (g) and water (ml) consumption of the pregnant rats during their gestation period were determined.

The blood glucose levels and skin fold thicknesses of the pregnant rats were determined periodically on days 6, 9, 12, 15, and 18 of the study. Blood glucose levels were measured from blood samples obtained from the lateral tail vein using a glucometer (OPTIMA). To assess any physiological oedema associated with pregnancy, the skin fold thicknesses and hind paw tarsal joint diameters of the rats were measured using a calliper (Holtain skinfold calliper) accurate to 0.2 mm. For the skin fold thickness measurements, only the skin and subcutaneous fat tissue (excluding muscle) were measured and recorded in millimeters ^(11)^.

***Abdominal skin thickness (ACT) measurement:*** The skin was grasped in the abdominal region parallel to the median line for a length of 3 cm, and the thickness was measured using a calliper.

***Shoulder (Cidago) skinfold thickness (SCT) measurement:*** The skin in the withers region was grasped by hand with the median line in the centre, and the thickness was measured using a calliper.

***Hind paw tarsal joint thickness (TJT) measurement:*** The hind paw was held, and the calliper was placed on the joint for the measurement. These measured values were calculated as percentages using the following formula; *ACT/SCT/TJT = (FM − M)/IM × 100 (FM:The final measured value, IM:The initial measured value)*

In the study, the rats were subjected to the Open Field Test, Social Preference Test, and Familiar Environment Test on the 19th day of pregnancy for the pregnancy and 10 days after birth for the offspring.

***Open field test:*** The open field test was used to determine the locomotor activity of the pregnant and juvenile rats^(12)^. For the test, the rats were placed in the centre of the open field test platform (Figure 1-b) and video recorded for five minutes. The recorded videos were evaluated for various behavioural activities, such as time spent in the centre (TSC), time spent in the periphery (TSP), number of entries and exits from the centre (NEEC), number of squares crossed (NSC), and number of defecations (ND).

***Social preference test:*** In the study, a modified version of the platform used by Kaidanovich-Beilin et al.^(13)^ was used (Figure 1-c). In this test, the preference of the pregnant rats towards a familiar rat (with whom they had been housed during pregnancy) and an unfamiliar rat was measured. After cleaning all compartments of the platform with alcohol, a rat from the same cage was placed in the first compartment, and an unfamiliar rat was placed in a plastic cage in the other compartment. The activity of the rat placed in the central compartment was recorded for 5 minutes. The recorded footage was analysed, and the familiar animal preference scores (FPS) and unfamiliar animal preference scores (UPS) of the groups were determined using the following formulae:


$$\begin{array}{cc} FPS=FT/TT\times 100 & UPS=UT/TT\times 100 \end{array}$$

(**TT:** Total time (s), **FT:** Time spent with the familiar animal (s), **UT:** Time spent with the unfamiliar animal (s)).

Similarly, the maternal preference scores for their own offspring (Own Offspring Preference Score or OOPS) and unfamiliar offspring (UOPS) were also calculated.


$$\begin{array}{cc} OOPS=OT/TT\times 100 & UOPS=UT/TT\times 100 \end{array}$$

(**TT:** Total time (s), **OT:** Time spent with own offspring (s), **UT:** Time spent with unfamiliar offspring (s))

***Light reflex test:*** A modified version of the platform used by Ergün and Taşkın for avian species was employed in this test^(14)^ (Figure 1-d). The platform consisted of 4 compartments measuring 20 × 20 × 33 cm, connected by passages, within enclosure of 50 × 50 × 35 cm. The interior of the compartments was illuminated using LED lights, adjusted to provide a light intensity of 150 ± 25 lux at the centre. This test was used to measure the Light Reflex (LR) of the pregnant rats towards the additional light colour they were exposed to during pregnancy. Additionally, this test was also applied to the 10-day-old offspring, measuring their LR towards the light colour experienced by their mothers. The first colour compartment to which the rats placed in the centre of the platform went within 5 minutes was considered as the preference.

***Behavioural observations:*** In this study, the gestational, maternal, and nursing behaviours of the rats were observed. Behavioural observations were conducted using the time sampling method by a single observer. Observations were performed four times a day (at 9:00 a.m., 12:00 noon, 4.00 p.m. and 8:p.m.) for five consecutive days. Behavioural observations were carried out according to the criteria specified in Chart 1 ^(15)^.

**Chart 1:** The ethograms used for the behavioural observations – Kirsehir, Turkiye, 2024.

Behaviour ethogram for pregnant rats		Maternal breastfeeding behaviour ethogram	
Behavioural criteria	Physical activity	Breastfeeding criteria	Physical activity
Nutrition	Eating and Drinking	Arched Breastfeeding (AB)	The mother’s arched-back suckling behaviour to Breastfeeding her offspring
Comfort	Body Stretching, Paw Stretching, Body Cleaning, and Paw-to-Head/Paw Cleaning	Blanket breastfeeding (BB)	The mother’s covering behaviour over her offspring, like a blanket, to facilitate breastfeeding.
Rest	Sleeping, Stretching	Passive breastfeeding (PB)	Non-breastfeeding situations and other situations
Social	Cage bar grasping and hanging, cage locomotion, and tail carriage		

### Ethical Aspects

The study was carried out in the Experimental Animals Unit of the Faculty of Medicine, Kirsehir Ahi Evran University, between May 2024 and June 2024, with the decision of the Local Ethics Committee for Animal Experiments dated January 19, 2024 and numbered 2/1.

### Statistical Analyses

The data obtained at the end of the study was analysed using the SPSS 22 statistical software package. Firstly, the effects of LED light on some behavioural and physiological characteristics of pregnant, maternal, and offspring rats were determined using one-way analysis of variance (ANOVA). In cases where significant differences were determined, the Duncan Test, one of the multiple comparison tests, was used to determine from which application(s) the difference originated^(16)^. Furthermore, p < 0.05 was accepted for all calculations in the study.

## RESULTS

The feed consumption amounts were similar in groups Y and C. The differences between these two groups and the other groups were significant (p < 0.05). The feed consumption amounts of the groups were 0.28 ± 0.03 g/BW(gr)/day for R, 0.25 ± 0.06 g/BW(gr)/day for groups Y and C, respectively, and 0.24 ± 0.04 g/BW(gr)/day for group B. There was a close relationship between the feed and water consumption of the rats. The daily water consumption amounts per BW of pregnant rats varied significantly (p < 0.05). In group R with the highest feed consumption, the water consumption was also the highest at 0.51 ± 0.06 ml/BW(gr)/day. The lowest water consumption was found in group C at 0.44 ± 0.04 ml/BW(gr)/day. The daily BW gain differences between the groups were significant (p < 0.05). The daily BW gain amounts of the groups were determined as follows: 4.60 ± 0.35 g/day for group R, 3.33 ± 0.58 g/day for group Y, 3.25 ± 0.88 g/day for group B, and 3.15 ± 0.51 g/day for group C (Table 1).

**Table 1.** Pregnant and offspring information in experimental rat groups– Kirsehir, Turkiye, 2024.

Information on the weight, daily physiological needs changes, and offspring of rat groups							
Groups	Body weight (g)	Weight gain (g/day)	Feed consumption (g/BW(g)/day)	Water consumption (mL/BW(g)/day)	Body weight loss due to birth (g)	Number of offspring (number)	Offspring weight (g)
C	193.21 ± 13.48	3.15 ± 0.51b	0.25 ± 0.06b	0.44 ± 0.04b	25.48 ± 1.62ab	11.33 ± 2.08a	5.51 ± 0.16ab
R	206.22 ± 15.72	4.60 ± 0.35a	0.28 ± 0.03a	0.51 ± 0.06a	27.91 ± 3.90a	12.66 ± 3.05a	4.91 ± 0.14c
B	198.50 ± 14.73	3.25 ± 0.88b	0.24 ± 0.04c	0.49 ± 0.02ab	26.28 ± 1.70ab	12.00 ± 3.60a	5.90 ± 0.01a
Y	208.42 ± 14.27	3.33 ± 0.58b	0.25 ± 0.06b	0.47 ± 0.01ab	23.12 ± 1.11b	12.00 ± 3.00a	5.30 ± 0.43bc
Values of Abdominal skin thickness (ACT), Shoulder (Cidago) skinfold thickness (SCT), and Hind paw tarsal joint hickness (TJT) for the groups							
**ACT**							
**Groups**	**Initial (mm)**			**11th day (+%)**	**20th day (-%)**		
C	13.66 ± 0.52ab			7.27 ± 0.36a	16.18 ± 3,30a		
R	13.00 ± 1.73b			7.09 ± 0.08a	16.42 ± 3.11a		
B	15.33 ± 0.57a			6.44 ± 0.38b	15.25 ± 2.88a		
Y	12.66 ± 0.57b			7.46 ± 0.39a	7.09 ± 0.08b		
**SCT**							
C	23.33 ± 0.57ab			7.18 ± 2.61a	8.86 ± 5.00a		
R	20.66 ± 0.57b			7.10 ± 2.56a	9.71 ± 2.44a		
B	24.33 ± 2.08ab			6.42 ± 2.09a	8.56 ± 4.33a		
Y	25.66 ± 3.21a			7.79 ± 0.17a	8.75 ± 5.44a		
**TJT**							
C	43.66 ± 1.15b			3.58 ± 1.11a	8.25 ± 3,30b		
R	42.66 ± 0.53b			3.78 ± 1.31a	14.94 ± 2.88a		
B	43.33 ± 1.15b			2.21 ± 0.18a	2.13 ± 3.11c		
Y	48.33 ± 1.52a			3.09 ± 1.02a	14.24 ± 0.08a		

*: Differences between means indicated by the same letter are not significant at the p < 0.05 level.

*: C; Control, R; Red, B; Blue, Y; Yellow.

The differences in weight losses due to birth among pregnant rats were significant (p < 0.05). The highest weight loss was measured in group R at 27.91 ± 3.90 g, while the lowest loss was recorded in group Y at 23.12 ± 1.11 g. The offspring weights were significant, with average weights being 5.90 ± 0.01 g for group B, 5.51 ± 0.16 g for group C, 5.30 ± 0.43 g for group Y, and 4.91 ± 0.14 g for group R, respectively (p < 0.05) (Table 1).

**Figure 2:**
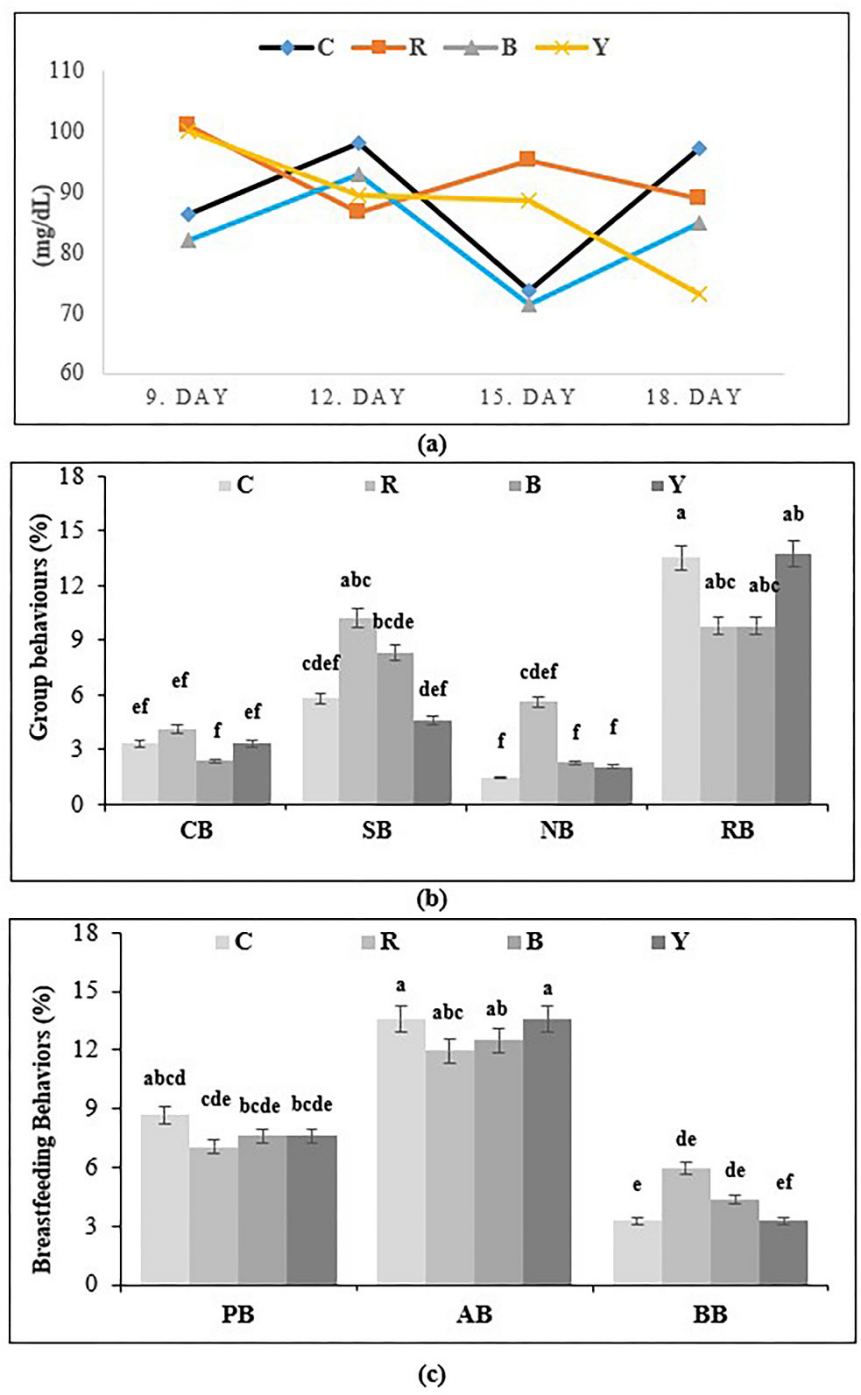
The blood glucose levels of pregnant rats and behavioural observations of rats. [a; Blood glucose (mg/dL) levels of pregnant rats (C; Control, R; Red, B; Blue, Y; Yellow), b; Time-dependent comfort behaviour, social behaviour, nutrition behaviour and resting behaviour values of the groups (%) (CB; Comfort Behaviour, SB; Social Behaviour, NB; Nutrition Behaviour, RB; Rest Behaviour), c; Breastfeeding behaviour values of the groups (%) (PB; Passive Breastfeeding, AB; Arched Breastfeeding, BB; Blanket Breastfeeding)]

The blood glucose levels of pregnant rats were measured every three days starting from the 9th day of pregnancy (Figure 2-a). The highest value among the groups was recorded on the 9th day of the study at 101.00 ± 6.55 mg/dL in group C, while the lowest value was detected on the 15th day of the study at 71.33 ± 2.32 mg/dL in group B. At the end of the 18th day, the highest value was determined in group C, and the lowest value was determined in group Y. Additionally, a consistent decrease was observed in group Y.

*: Differences between means indicated by the same letter are not significant at the p < 0.05 level.

In the study, ACT, SCT and TJT were measured during the initial, mid, and final periods to detect physiological oedema that may occur in pregnant rats (Table 1). Skinfold thickness increased until the 11th day of pregnancy, but in later measurements, a decrease was observed in skinfold thickness values. The lowest values were detected under blue light on the 11th day of pregnancy. Yellow light had an oedema-promoting effect. Measurements taken on the 20th day of pregnancy indicated that red light had a more significant oedema-reducing effect than did other light.

In the study, behavioural tests including the Open Field Test, Social Preference Test, and Light Reflex Tests were applied to pregnant rats on the 19th day and to the newborn on the 10th day. The Open Field Test was conducted to determine the locomotor activities of the pregnant rats and their offspring. Characteristics such as the TSC, TSP, NEEC, NSC, and the ND were recorded (Table 2). The values for the groups were significant at the p < 0.05 level.

**Table 2:** Open field, social preference, light reflex test table for pregnant and offspring rats, and the table for mothers’ offspring preference – Kirsehir, Turkiye, 2024.

Open field test table				
Pregnant rats				
Groups	TSC *(sec)*	TSP *(sec)*	NEEC *(number)*	NSC *(number)*	ND *(number)*
C	2.50 ± 2.12b	296.50 ± 0.71a	2.00 ± 1.00b	13.33 ± 2.30d	1.33 ± 0.57ab
R	18.50 ± 3.53a	277.50 ± 3.53b	4.00 ± 1.73a	57.33 ± 2.30a	00.0 ± 0.00b
B	5.50 ± 2.12b	294.50 ± 2.12a	4.33 ± 0.57a	37.66 ± 2.88bc	0.33 ± 0.57b
Y	1.50 ± 0.70b	298.50 ± 0.70a	1.66 ± 1.15ab	44.66 ± 6.35b	2.00 ± 1.00a
**Offspring rats**					
C	13.66 ± 5.13c	285.33 ± 3.51a	2.33 ± 0.57a	24.00 ± 2.00b	0.00 ± 0.00
R	43.33 ± 5.13a	256.66 ± 5.13c	2.66 ± 1.15a	67.00 ± 2.08a	0.00 ± 0.00
B	25.33 ± 4.04b	274.66 ± 4.04b	3.33 ± 2.30a	17.33 ± 2.08c	0.00 ± 0.00
Y	22.66 ± 1.15b	276.56 ± 1.15b	2.00 ± 1.00ab	23.66 ± 1.52b	0.00 ± 0.00
**Social preference and light reflex test table**					
**Pregnant rats**					
**Groups**	**FPS (%)**		**UPS (%)**		**LR (%)**	
C	7.00 ± 2.82c		65.10 ± 3.53a		16.15 ± 0.25c	
R	81.66 ± 2.35a		7.95 ± 1.90b		33.15 ± 0.16b	
B	13.65 ± 0.49b		73.90 ± 3.74a		50.01 ± 0.40a	
Y	12.30 ± 2.40bc		72.65 ± 3.74a		16.15 ± 0.25c	
**Offspring rats**					
C	2.03 ± 0.64c		88,32 ± 4.00a		33.17 ± 0.16b	
R	58.14 ± 2.24a		27.22 ± 3.47d		62.25 ± 3.81a	
B	18.69 ± 5.25b		59.81 ± 5.56c		16.25 ± 0.35c	
Y	1.84 ± 0.32c		78.14 ± 2.50b		16.32 ± 0.33c	
**Mother’s offspring preference table**			**OOPS (%)**		**UOPS (%)**
C			30.52 ± 3.10b		56.11 ± 5.50a
R			64.16 ± 1.01a		31.66 ± 2.35c
B			29.16 ± 0.39b		42.31 ± 1.43b
Y			30.27 ± 0.38b		53.30 ± 3.93a

*: Differences between means indicated by the same letter are not significant at the p < 0.05 level.

*: C; Control, R; Red, B; Blue, Y; Yellow, TSC; Time Spent in the Center, TSP; Time Spent in the Periphery, NEEC; Number of Entries and Exits from the Center, NSC; Number of Squares Crossed, ND; Number of Defecations, FPS; Familiar animal Preference Scores, UPS; Unfamiliar animal Preference Scores, LR; Light Reflex, OOPS; Own Offspring Preference Score, UOPS; Unfamiliar Offspring Preference Score

The time spent in the centre (TSC) by pregnant rats was 18.50 ± 3.53 second (sec) for group R, 5.50 ± 2.12 sec for group B, 2.50 ± 2.12 sec for group C, and 1.50 ± 0.70 sec for group Y. The TSP was 298.50 ± 0.70 sec for group Y, 296.50 ± 0.71 sec for group C, 294.50 ± 2.12 sec for group B, and 277.50 ± 3.53 sec for group R. Similarly, for the offspring, the TSC was 43.33 ± 5.13 sec for group R, 25.33 ± 4.04 sec for group B, 22.66 ± 1.15 sec for group Y, and 13.66 ± 5.13 sec for group C. The TSP values for the offspring were measured at 285.33 ± 3.51 sec for group C, 276.56 ± 1.15 sec for group Y, 274.66 ± 4.04 sec for group B, and 256.66 ± 5.13 sec for group R.

In pregnant rats, the NEEC was the highest in group B at 4.33 ± 0.57 and lowest in group Y at 1.66 ± 1.15. The NSC was 57.33 ± 2.30 in group R, 44.66 ± 6.35 in group Y, 37.66 ± 2.88 in group B, and 13.33 ± 2.30 in group C. In the offspring, this value was the highest for NEEC in group B, while the highest NSC was in group R. The values obtained in the offspring were similar to those in the pregnant rats.

In the study, significant differences were between the rates of FPS and UPS in pregnant rats at the p < 0.05 level (Table 2). The highest FPS was recorded at 81.66 ± 2.35% in group R, while the lowest value was in group C at 7.00 ± 2.82%. In the groups, the UPS was 73.90 ± 3.74% in group B, 72.65 ± 3.74% in group Y, 65.10 ± 3.53% in group C and 7.95 ± 1.90% in group R, respectively.

In the study, the preferences of offspring rats for their mothers and unfamiliar adults were investigated. Significant differences were determined between the groups (p < 0.05). The offspring of rats exposed to red light preferred to spend more time with their mothers, with a preference of 58.14 ± 2.24%. In contrast, the offspring from other colour groups tended to favour unfamiliar individuals. The preferences for LR among the study groups were significantly different at the p < 0.05 level. In pregnant rats, the highest light preference was in the blue group at 50.01 ± 0.40%, while the lowest preference was observed in groups C and Y at 16.15 ± 0.25%. For the offspring, the highest preference value was calculated in group R at 62.25 ± 3.81%, and the lowest was in group B, at 16.25 ± 0.35% (Table 2).

In the study, the effects of the light applied to pregnant rats on offspring preference after birth were investigated (Table 2). Significant differences were determined between the groups (p < 0.05). The highest rate of OOPS was recorded in group R at 64.16 ± 1.01%, while the highest UOPS was in the control group at 56.11 ± 5.50%. Significant differences were also observed in behavioural criteria among the groups (p < 0.05) (Figure 2-b). The highest comfort levels in social and feeding behaviour assessments were identified in R, while the highest resting behaviour was observed in C.

In the study, the observations made throughout the day revealed that the most frequent behaviours observed were the blanket, passive, and arched breastfeeding behaviours, in the given order. There were also differences in breastfeeding behaviours related to light wavelength, with the highest blanket breastfeeding observed under red light (Figure 2-c).

## DISCUSSION

Environmental light and light colour are among the significant environmental factors that directly affect pregnancy, motherhood, and infancy^(17)^. In the study, the highest feed and water consumption, as well as the amount of weight gain, were observed in group R. The lowest feed consumption was noted in group B, while the least water consumption and weight gain were in group C. It is believed that the differences observed in feed and water consumption and daily weight gain are influenced by the applied light wavelengths. The additional red light applied during pregnancy increased feed and water consumption, leading to weight gain. Wren-Dail et al.^(18)^ reported in 2016 that male Sprague-Dawley rats aged 4–5 weeks had higher feed and water consumption and weight gain when exposed to red light. Our findings are consistent with this result. In many studies, it has been indicated that light wavelength affects individuals’ motivation to eat, increases appetite and eating speed, and also influences unhealthy feed choices^(19)^. In humans, excessive exposure to light affects BW, with morning exposure leading to lower BW and late exposure resulting in higher BW^(20)^.

In the study, the average offspring weights were determined to be between 5.90 ± 0.01 g and 4.91 ± 0.14 g. The highest value was observed in the group exposed to blue light, while the lowest value was observed in the group exposed to red light. The number of offspring was similar in all the groups. Based on the findings, it is suggested that blue light may have a weight-increasing effect on offspring development, whereas red light may have a decreasing effect. Balcıoğlu and Özdamar^(21)^ reported that the birth weights of offspring rats ranged between 5.65 ± 0.11 g and 5.02 ± 0.13 g. The values determined in the present study are similar to those reported by Balcıoğlu and Özdamar (2020).

The highest blood glucose level among the groups was observed in group C on day 9 of the study, while the lowest was in group B on day 15. Throughout the study, a continuous decrease in blood glucose levels was noted in the rats of group Y. Cheung et al.^(22)^ reported that light had an effect on glucose metabolism. The identification of differences in blood glucose levels among the groups with varying light applications is consistent with this information.

Exposure to environmental stress during pregnancy can affect the maturation of developing offspring, potentially leading to changes in learning, behaviours, and emotions later in life^(23)^. In the open field test, a decrease in the distance travelled and visits to the centre is an indicator of stress^(24)^. The highest number of defecations was observed in group Y, while no defecation, which is a sign of stress and fear, was noted in the pregnant rats and offspring in group R. The results we obtained support the findings of the TSC and TSP results.

Animals prefer socialization over being alone and are more inclined to welcome new individuals into their environment. This leads them to favour unfamiliar peers over familiar ones^(25)^. In the study, it was observed that the applied light wavelength affected the differences between the FPS and UPS ratios of pregnant rats which can be explained by the fact that red light increases FPS in pregnant rats, while blue light increases UPS, contrary to this.

Olfactory bonds established with the mother during early development are crucial for healthy social development. Later stages of life are influenced by the positive and beneficial relationships formed during this period. Numerous studies have been conducted to identify the olfactory bonds between mothers and their offspring^(26)^. In our study, the offspring of rats exposed to red light preferred to spend more time alongside their mothers compared to others.

In the study, the highest rate of OOPS was determined in group R, while the highest rate of UOPS was observed in group C, suggesting that exposure to red light during pregnancy may enhance the feelings of maternal attachment towards the offspring. Significant differences were identified in behavioural criteria between the groups, which=may stem from both the direct effects of the light wavelength on behaviours and its secondary effects through neural pathways^(27)^. The highest comfort, social, and feeding behaviour assessments were determined in group R, while the highest resting behaviour was observed in group C. Maternal stress during pregnancy leads to a reduction in social behaviours. The observation of the highest social behaviour in group R indicates the stress-reducing effect of red light, consistent with Laviola et al.’s findings^(28)^. Similarly, in a study conducted on primates, it was reported that red light promoted active behaviour in primates, while blue light led to a decrease in activity^(29)^. Additionally, the behavioural effects of light on humans are often observed as behavioural dysfunction, depression, and psychological disorders^(30)^. In the study, the most frequently observed behaviours during the day were arched, passive, and blanket breastfeeding behaviours.

## CONCLUSION

It was concluded that the additional red light applied during pregnancy had a weight-increasing effect on pregnant rats, while it reduced birth weight in the offspring and also had a diuretic effect. Blue light had a weight-increasing effect on the offspring, alongside its oedema-preventing effects. Yellow light resulted in a consistent decrease in glucose metabolism during pregnancy. Furthermore, red light increased comfort, social, and feeding behaviours in pregnant rats, reduced stress for both pregnant rats and offspring, enhanced the preference for familiar individuals, increased feelings of motherhood and attachment towards the offspring, and positively affected the ability to distinguish the mother’s scent and the attachment feelings towards her. Furthermore, this study is expected to provide insights into the subsequent investigations to be conducted on these effects in other mammals, such as rats. More experimental studies are needed to assess the effects of different coloured LED lighting on the physiological and behavioural characteristics of pregnant rats, mothers, and newborn.

## DATA AVAILABILITY

Data will be made available on reasonable request.
