# Supplementary material for: Investigation of effects of different colour led lighting application to pregnant rats on mother and offspring
Source: Rev Esc Enferm USP. 2025 Jul 7;59:e20240398. doi: 10.1590/1980-220X-REEUSP-2024-0398en (PMC12258052; doi:10.1590/1980-220X-REEUSP-2024-0398en)
Supplement: Translation

# Translation: Investigación de los efectos de la aplicación de iluminación led de diferentes colores a ratas gestantes sobre la madre y la cría

**DOI:** 10.1590/1980-220X-REEUSP-2024-0398es

**Authors:** Halime Aydemir, Fatma Ergün

**Affiliations:** 1 Kirsehir Ahi Evran University, Faculty of Health Sciences, Department of Midwifery, Kirsehir, Türkiye.; 2 Kirsehir Ahi Evran University, Faculty of Health Sciences, Department of Nutrition and Dietetics, Kirsehir, Türkiye.

**Keywords:** Rata, Luz, Embarazo, Luz roja, Crías

## Abstract

**Objetivo:**

En el estudio se investigaron los efectos de la iluminación LED de diferentes colores sobre las características fisiológicas y de comportamiento de ratas gestantes, ratas madre y sus crías, utilizando un modelo experimental consistente en ratas primíparas (que dan a luz por primera vez).

**Método:**

En el estudio se incluyeron 24 ratas gestantes (Wistar Albino) en 4 grupos, cada uno de los cuales incluía. Las ratas se colocaron en 4 armarios de luz (blanca, roja, azul y amarilla). Durante la gestación y el posparto se evaluaron el peso corporal de las ratas gestantes/madres y sus crías, el aumento de peso de las ratas gestantes, el consumo diario de pienso y agua, los niveles de glucosa en sangre, el grosor de la piel y las características de comportamiento de las ratas gestantes y sus crías.

**Resultados:**

El consumo de alimento y agua fue mayor en el grupo Rojo (0,51 ± 0,06 ml/PC (g)/día). Se determinaron diferencias significativas entre los grupos de ratas en cuanto a sus niveles de glucosa en sangre, el grosor de la piel y los resultados de las características de comportamiento de las ratas gestantes y sus crías (p < 0,05).

**Conclusiones:**

La luz roja tuvo un efecto de aumento de peso en las ratas gestantes, mientras que la luz azul tuvo un efecto de prevención del edema y un efecto de aumento de peso en las crías.

## INTRODUCCIÓN

La mejora de la salud materna y neonatal y la reducción de la morbimortalidad materna y neonatal revisten gran importancia para la salud de las mujeres embarazadas^(1)^. El embarazo se considera un periodo crítico para la salud materna y neonatal.

La atención basada en directrices contrastadas mejora la calidad de la asistencia^(2)^. Las recomendaciones de la Organización Mundial de la Salud (OMS) para una experiencia positiva del embarazo en la atención prenatal incluyen una nutrición sana y ejercicio, suplementos diarios de hierro y ácido fólico, suplementos diarios de calcio y limitar la ingesta de cafeína por encima de 300 mg al día. Las recomendaciones para las embarazadas incluyen el diagnóstico de la anemia, la ecografía precoz, la prueba de tolerancia a la glucosa, la detección del consumo de tabaco y el tabaquismo pasivo, la violencia de pareja, la bacteriuria asintomática con 7 días de antibióticos, la inmunoglobulina anti-D y el tratamiento preventivo antihelmíntico. La OMS también recomienda que toda embarazada disponga de información sanitaria propia, vacunación antitetánica, número de visitas prenatales y recomendaciones para prevenir las molestias comunes del embarazo (hiperémesis gravídica, acidez, calambres, dolor de espalda y pélvico, estreñimiento, varices y edemas)^(3)^. Estas recomendaciones tienen por objeto garantizar una evolución sana del embarazo.

Sin embargo, incluso con un embarazo sano monitoreado, hay factores inevitables en la vida diaria, uno de los cuales es la luz.

La luz es una fuente indispensable para mantener nuestra vida mientras estamos despiertos^(4)^. Durante el embarazo, la mujer se enfrenta a los efectos de los sistemas de iluminación en los procesos de gestación, parto y posparto. Aunque las luces LED utilizadas en la iluminación actual se tienen en cuenta a la hora de monitorear y cuidar de acuerdo con las recomendaciones de la OMS, son un factor que puede afectar al proceso del embarazo. Dadas las necesidades de la vida humana, es una realidad que resulta difícil eliminar los efectos de la iluminación en las condiciones actuales.

Se ha informado de que las luces LED azules afectan a los niveles de bilirrubina y acortan el tiempo de tratamiento en la fototerapia^(5)^. La fototerapia se ha utilizado durante muchos años como método fiable y eficaz en el tratamiento de la ictericia neonatal^(6)^. Este estudio experimental se diseñó para monitorear y evaluar los efectos de las luces LED en ratas. Se espera que el estudio arroje luz sobre los estudios acerca de cómo afectan las luces LED a otros mamíferos como las ratas.

## MÉTODO

En el estudio se utilizaron 24 ratas hembras primíparas (preñadas por primera vez) (Wistar Albino) de 14−15 semanas de edad (peso corporal [PC] 200 ± 15 g), con características morfológicas similares, criadas en la Unidad de Animales Experimentales de la Facultad de Medicina de la Universidad Kirsehir Ahi Evran, Turquía.

Todas las fases del estudio se llevaron a cabo en un entorno con una temperatura de 22 ± 2 °C, una humedad relativa del 35 ± 10% y un nivel de ruido de 35 ± 10 decibelios (dB)^(7)^. El entorno del estudio estaba iluminado con luz blanca en un ciclo de 12 horas de luz/12 horas de oscuridad. Para que las ratas hembras quedaran preñadas, se colocaron 24 ratas hembras por parejas en 12 jaulas de apareamiento. En cada jaula se colocó una rata macho de 18−20 semanas (2 hembras/1 macho). Después de que las ratas macho permanecieran en las jaulas durante 6 días (el ciclo estral de las ratas es de 4−5 días), se sacaron de las jaulas^(8)^. A continuación, las ratas hembras se dividieron en 4 grupos, cada uno con 6 ratas de forma homogénea. Las 6 ratas de cada grupo se colocaron en 3 jaulas de cría (425*265*150/800 cm^2^)^(9)^ que se colocaron en 4 armarios de luz especialmente preparados (Blanco, Rojo, Azul, Amarillo) para el estudio. Las cabinas estaban hechas de poliestireno expandido blanco (TS7316- EN13163) con unas dimensiones de 100 × 50 × 50 cm, con las partes frontales abiertas e impermeables a la luz. Se instalaron fuentes de luz LED en el techo de los armarios para proporcionar una intensidad luminosa de 150 ± 25 lux en el suelo; además del ciclo de 12 horas de luz/12 horas de oscuridad, los armarios se iluminaron con luces blancas (400–740 nm), rojas (635–700 nm), azules (450–490 nm) y amarillas (560–590 nm) durante 4 horas^(10)^. La aplicación de luz adicional se llevó a cabo entre las 19:00 y las 23:00 horas siguientes al periodo de luz (Figura 1-a).

**Figura 1:** Armarios iluminados con luces LED de distintos colores y plataformas de ensayo de análisis del comportamiento (a; jaulas de cría, b; plataforma de ensayo en campo abierto, c; plataforma de ensayo de preferencia social, d; plataforma de ensayo de reflejo luminoso).

La intensidad de la luz y los periodos de duración utilizados se inspiraron en los patrones de iluminación típicos del hogar y la oficina (entorno iluminado desde el atardecer hasta la medianoche). Además, la iluminación blanca continua utilizada habitualmente en entornos domésticos y de oficina se designó como grupo de control en el estudio. La intensidad luminosa dentro de los armarios se monitoreó cada 3 días utilizando un luxómetro digital (Extech 5-in-1 Environmental Meter EN300).

Durante los periodos de gestación y posparto, se midió el peso corporal de las ratas gestantes/madres y de sus crías utilizando una báscula digital (NECK WT-NF Precision Scale) con una precisión de 0,01 unidades, y se calcularon las ganancias diarias de peso corporal (g/día). Además, se determinó el consumo diario de alimento (g) y agua (ml) de las ratas gestantes durante el periodo de gestación.

Los niveles de glucosa en sangre y el grosor de los pliegues cutáneos de las ratas gestantes se determinaron periódicamente los días 6, 9, 12, 15 y 18 del estudio. Los niveles de glucosa en sangre se midieron a partir de muestras de sangre obtenidas de la vena lateral de la cola utilizando un glucómetro (OPTIMA). Para evaluar cualquier edema fisiológico asociado al embarazo, se midieron el grosor de los pliegues cutáneos y los diámetros de la articulación tarsal de la pata trasera de las ratas utilizando un calibrador (Holtain skinfold calliper) con una precisión de 0,2 mm. Para las mediciones del grosor de los pliegues cutáneos, sólo se midieron y registraron en milímetros la piel y el tejido adiposo subcutáneo (excluidos los músculos)^(11)^.

***Medición del grosor de la piel abdominal (ACT)***: Se agarró la piel en la región abdominal paralelamente a la línea mediana durante una longitud de 3 cm y se midió el grosor con un calibrador.

***Medición del espesor del pliegue cutáneo del hombro (Cidago) (SCT)***: Se tomó con la mano la piel de la región de la cruz, con la línea mediana en el centro, y se midió el grosor con un calibrador.

***Medición del grosor de la articulación tarsal de la pata trasera (TJT)***: Se sujetó la pata trasera y se colocó el calibrador en la articulación para la medición. Estos valores medidos se calcularon como porcentajes mediante la siguiente fórmula: *ACT/SCT/TJT = (FM − IM)/IM × 100 (FM: valor medido final, IM: valor medido inicial)*.

En el estudio, las ratas fueron sometidas al Test de Campo Abierto, al Test de Preferencia Social y al Test de Ambiente Familiar el día 19 de gestación para las gestantes y 10 días después del nacimiento para las crías.

***Prueba de campo abierto***: La prueba de campo abierto se utilizó para determinar la actividad locomotora de las ratas gestantes y de las crías^(12)^. Para la prueba, las ratas se colocaron en el centro de la plataforma de prueba de campo abierto (Figura 1-b) y se grabaron en vídeo durante cinco minutos. En los vídeos grabados se evaluaron diversas actividades conductuales, como el tiempo pasado en el centro (TSC), el tiempo pasado en la periferia (TSP), el número de entradas y salidas del centro (NEEC), el número de cuadrados cruzados (NSC) y el número de defecaciones (ND).

***Prueba de preferencia social***: En el estudio se utilizó una versión modificada de la plataforma empleada por Kaidanovich-Beilin et al.^(13)^ (Figura 1-c). En esta prueba, se midió la preferencia de las ratas gestantes hacia una rata familiar (con la que habían estado alojadas durante la gestación) y una rata desconocida. Tras limpiar todos los compartimentos de la plataforma con alcohol, se colocó una rata de la misma jaula en el primer compartimento y una rata desconocida en una jaula de plástico en el otro compartimento. Se grabó la actividad de la rata colocada en el compartimento central durante 5 minutos. Se analizaron las imágenes grabadas y se determinaron las puntuaciones de preferencia por animales familiares (FPS) y las puntuaciones de preferencia por animales desconocidos (UPS) de los grupos mediante las siguientes fórmulas:


$$\begin{array}{cc} FPS=FT/TT\times 100 & UPS=UT/TT\times 100 \end{array}$$

(**TT**: Tiempo total (s), **FT**: Tiempo pasado con el animal familiar (s), **UT**: Tiempo pasado con el animal desconocido (s)).

Del mismo modo, también se calcularon las puntuaciones de preferencia materna por las crías propias (Own Offspring Preference Score u OOPS) y las crías desconocidas (UOPS).


$$\begin{array}{cc} OOPS=OT/TT\times 100 & UOPS=UT/TT\times 100 \end{array}$$

(**TT**: Tiempo total (s), **OT**: Tiempo dedicado a las crías propias (s), **UT**: Tiempo pasado con crías desconocidas (s))

***Prueba de reflejos luminosos***: En esta prueba se empleó una versión modificada de la plataforma utilizada por Ergün y Taşkın para especies aviares^(14)^ (Figura 1-d). La plataforma constaba de 4 compartimentos de 20 × 20 × 33cm, conectados por pasillos, dentro de un recinto de 50 × 50 × 35cm. El interior de los compartimentos se iluminó con luces LED, ajustadas para proporcionar una intensidad luminosa de 150 ± 25 lux en el centro. Esta prueba se utilizó para medir el reflejo luminoso (RL) de las ratas gestantes hacia el color de luz adicional al que estaban expuestas durante la gestación. Además, esta prueba también se aplicó a las crías de 10 días, midiendo su RL hacia el color de luz experimentado por sus madres. Se consideró como preferencia el primer compartimento de color al que se dirigieron las ratas colocadas en el centro de la plataforma en un plazo de 5 minutos.

***Observaciones del comportamiento***: En este estudio se observaron los comportamientos de gestación, maternidad y lactancia de las ratas. Las observaciones del comportamiento se realizaron mediante el método de muestreo temporal por un solo observador. Las observaciones se realizaron cuatro veces al día (a las 9.00 h, a las 12.00 h, a las 16.00 h y a las 20.00 h) durante cinco días consecutivos. Las observaciones del comportamiento se realizaron según los criterios especificados en el Cuadro 1 ^(15)^.

**Cuadro 1:** Etogramas utilizados para las observaciones de comportamiento - Kirsehir, Turquía, 2024.

Etograma del comportamiento de las ratas preñadas		Etograma del comportamiento materno en materia de lactancia materna	
Criterios de comportamiento	Actividad física	Criterios de lactancia	Actividad física
Nutrición	Comida y bebida	Amamantamiento en arco (AB)	El comportamiento de succión arqueado hacia atrás de la madre para amamantar a su cría
Confort	Estiramiento del cuerpo, estiramiento de las patas, limpieza del cuerpo y limpieza de las patas y la cabeza	Amamantamiento con manta (BB)	Comportamiento de la madre que cubre a su cría, como una manta, para facilitar la lactancia.
Descanso	Dormir, Estirarse	Lactancia materna pasiva (PB)	Situaciones de no lactancia y otras situaciones
Social	Agarre y suspensión de la barra de la jaula, locomoción en la jaula y transporte de la cola		

### Aspectos Éticos

El estudio se llevó a cabo en la Unidad de Animales Experimentales de la Facultad de Medicina de la Universidad Kirsehir Ahi Evran, entre mayo de 2024 y junio de 2024, con la decisión del Comité Local de Ética para Experimentos con Animales de fecha 19 de enero de 2024 y número 2/1.

### Análisis Estadísticos

Los datos obtenidos al final del estudio se analizaron utilizando el paquete de software estadístico SPSS 22. En primer lugar, se determinaron los efectos de la luz LED sobre algunas características conductuales y fisiológicas de las ratas gestantes, maternas y crías mediante un análisis de varianza de una vía (ANOVA). En los casos en que se determinaron diferencias significativas, se utilizó la prueba de Duncan, una de las pruebas de comparación múltiple, para determinar de qué aplicación o aplicaciones procedía la diferencia^(16)^. Además, se aceptó p < 0,05 para todos los cálculos del estudio.

## RESULTADOS

Las cantidades de consumo de pienso fueron similares en los grupos Y y C. Las diferencias entre estos dos grupos y los demás fueron significativas (p < 0,05). Las cantidades de consumo de pienso de los grupos fueron de 0,28 ± 0,03 g/PC (gr)/día para R, 0,25 ± 0,06 g/PC (gr)/día para los grupos Y y C, respectivamente, y 0,24 ± 0,04 g/PC (gr)/día para el grupo B. Existía una estrecha relación entre el consumo de pienso y de agua de las ratas. Las cantidades diarias de consumo de agua por peso corporal de las ratas gestantes variaron significativamente (p < 0,05). En el grupo R, con el mayor consumo de pienso, el consumo de agua fue también el más elevado, con 0,51 ± 0,06 ml/PC(gr)/día. El menor consumo de agua se encontró en el grupo C con 0,44 ± 0,04 ml/PC(gr)/día.

Las diferencias de ganancia diaria de peso corporal entre los grupos fueron significativas (p < 0,05). Las cantidades diarias de ganancia de peso de los grupos se determinaron como sigue: 4,60 ± 0,35 g/día para el grupo R, 3,33 ± 0,58 g/día para el grupo Y, 3,25 ± 0,88 g/día para el grupo B y 3,15 ± 0,51 g/día para el grupo C (Tabla 1).

**Tabla 1:** Información sobre la preñez y la descendencia en los grupos de ratas experimentales - Kirsehir, Turquía, 2024.

Información sobre el peso, los cambios en las necesidades fisiológicas diarias y las crías de los grupos de ratas							
Grupos	Peso corporal (g)	Ganancia de peso (g/día)	Consumo de alimento (g/BW(g)/día)	Consumo de agua (mL/BW(g)/día)	Pérdida de peso debida al parto (g)	Número de crías	Peso de las crías (g)
C	193.21 ± 13.48	3.15 ± 0.51b	0.25 ± 0.06b	0.44 ± 0.04b	25.48 ± 1.62ab	11.33 ± 2.08a	5.51 ± 0.16ab
R	206.22 ± 15.72	4.60 ± 0.35a	0.28 ± 0.03a	0.51 ± 0.06a	27.91 ± 3.90a	12.66 ±3.05a	4.91 ± 0.14c
B	198.50 ± 14.73	3.25 ± 0.88b	0.24 ± 0.04c	0.49 ± 0.02ab	26.28 ± 1.70ab	12.00 ± 3.60a	5.90 ± 0.01a
Y	208.42 ± 14.27	3.33 ± 0.58b	0.25 ± 0.06b	0.47 ± 0.01ab	23.12 ± 1.11b	12.00 ± 3.00a	5.30 ± 0.43bc
Valores de Espesor de la piel abdominal (ACT), Espesor del pliegue cutáneo del hombro (Cidago) (SCT) y Espesor de la articulación tarsal de la pata trasera (TJT) de los grupos							
**ACT**							
**Grupos**	**Inicial (mm)**			**Día 11(+%)**	**Día 20 (−%)**		
C	13.66 ± 0.52ab			7.27 ± 0.36a	16.18 ± 3,30a		
R	13.00 ± 1.73b			7.09 ± 0.08a	16.42 ± 3.11a		
B	15.33 ± 0.57a			6.44 ± 0.38b	15.25 ± 2.88a		
Y	12.66 ± 0.57b			7.46 ± 0.39a	7.09 ± 0.08b		
**SCT**							
C	23.33 ± 0.57^ab^			7.18 ± 2.61^a^	8.86 ± 5.00^a^		
R	20.66 ± 0.57^b^			7.10 ± 2.56^a^	9.71 ± 2.44^a^		
B	24.33 ± 2.08^ab^			6.42 ± 2.09^a^	8.56 ± 4.33^a^		
Y	25.66 ± 3.21^a^			7.79 ±0.17^a^	8.75 ±5.44^a^		
**TJT**							
C	43.66 ± 1.15b			3.58 ± 1.11a	8.25 ± 3,30b		
R	42.66 ± 0.53b			3.78 ±1.31a	14.94 ± 2.88a		
B	43.33 ± 1.15b			2.21 ± 0.18a	2.13 ± 3.11c		
Y	48.33 ± 1.52a			3.09 ± 1.02a	14.24 ± 0.08a		

*: Las diferencias entre las medias indicadas con la misma letra no son significativas al nivel p < 0,05.

*: C; Control, R; Rojo, B; Azul, Y; Amarillo

Las diferencias en las pérdidas de peso debidas al parto entre las ratas gestantes fueron significativas (p < 0,05). La mayor pérdida de peso se midió en el grupo R con 27,91 ± 3,90 g, mientras que la menor se registró en el grupo Y con 23,12 ± 1,11 g. Los pesos de las crías fueron significativos, con pesos medios de 5,90 ± 0,01 g para el grupo B, 5,51 ± 0,16 g para el grupo C, 5,30 ± 0,43 g para el grupo Y y 4,91 ± 0,14 g para el grupo R, respectivamente (p < 0,05) (Tabla 1).

Los niveles de glucosa en sangre de las ratas gestantes se midieron cada tres días a partir del 9° día de gestación (Figura 2-a). El valor más alto entre los grupos se registró el 9º día del estudio con 101,00 ± 6,55 mg/dL en el grupo C, mientras que el valor más bajo se detectó el 15º día del estudio con 71,33 ± 2,32 mg/dL en el grupo B. Al final del 18º día, el valor más alto se determinó en el grupo C, y el valor más bajo se determinó en el grupo Y. Además, se observó un descenso constante en el grupo Y.

**Figura 2:** Niveles de glucosa en sangre de ratas gestantes y observaciones del comportamiento de las ratas. [a; Niveles de glucosa en sangre (mg/dL) de ratas gestantes (C; Control, R; Rojo, B; Azul, Y; Amarillo), b; Valores de comportamiento de confort, comportamiento social, comportamiento de nutrición y comportamiento de reposo de los grupos en función del tiempo (%) (CB; Comportamiento de confort, SB; Comportamiento social, NB; Comportamiento de nutrición, RB; Comportamiento de reposo), c; Valores de comportamiento de amamantamiento de los grupos (%) (PB; Amamantamiento pasivo, AB; Amamantamiento arqueado, BB; Amamantamiento con manta)].

*: Las diferencias entre medias indicadas con la misma letra no son significativas al nivel p < 0,05.

En el estudio se midieron el ACT, el SCT y el TJT durante los periodos inicial, medio y final para detectar el edema fisiológico que puede producirse en las ratas gestantes (Tabla 1). El grosor de los pliegues cutáneos aumentó hasta el undécimo día de gestación, pero en las mediciones posteriores se observó una disminución de los valores de grosor de los pliegues cutáneos. Los valores más bajos se detectaron con luz azul el 11º día de gestación. La luz amarilla tuvo un efecto promotor del edema. Las mediciones realizadas el vigésimo día de embarazo indicaron que la luz roja tenía un efecto reductor del edema más significativo que las demás luces.

En el estudio se aplicaron a las ratas gestantes, el día 19, y a las recién nacidas, el día 10, pruebas de comportamiento que incluían la Prueba de Campo Abierto, la Prueba de Preferencia Social y las Pruebas de Reflejos Lumínicos. El Test de Campo Abierto se realizó para determinar las actividades locomotoras de las ratas gestantes y sus crías. Se registraron características como el TSC, el TSP, el NEEC, el NSC y el ND (Tabla 2). Los valores de los grupos fueron significativos al nivel p<0,05.

**Tabla 2:** Tablas de Campo abierto, preferencia social, pruebas de reflejo de luz para ratas gestantes y crías, y la tabla de preferencia de las crías de las madres - Kirsehir, Turquía, 2024.

Tabla de pruebas de campo abierto					
Ratas preñadas					
Grupos	TSC (*sec*)	TSP (*sec*)	NEEC (*numero*)	NSC (*numero*)	ND (*numero*)
C	2.50 ± 2.12^b^	296.50 ± 0.71^a^	2.00 ± 1.00^b^	13.33 ± 2.30^d^	1.33 ± 0.57^ab^
R	18.50 ± 3.53^a^	277.50 ± 3.53^b^	4.00 ± 1.73^a^	57.33 ± 2.30^a^	00.0 ± 0.00^b^
B	5.50 ± 2.12^b^	294.50 ± 2.12^a^	4.33 ± 0.57^a^	37.66 ± 2.88^bc^	0.33 ± 0.57^b^
Y	1.50 ± 0.70^b^	298.50 ± 0.70^a^	1.66 ±1.15^ab^	44.66 ± 6.35^b^	2.00 ± 1.00^a^
**Crías de ratas**					
C	13.66 ± 5.13^c^	285.33 ±3.51^a^	2.33 ± 0.57^a^	24.00 ± 2.00^b^	0.00 ± 0.00
R	43.33 ± 5.13^a^	256.66 ± 5.13^c^	2.66 ± 1.15^a^	67.00 ± 2.08^a^	0.00 ± 0.00
B	25.33 ± 4.04^b^	274.66 ± 4.04^b^	3.33 ± 2.30^a^	17.33 ± 2.08^c^	0.00 ± 0.00
Y	22.66 ± 1.15^b^	276.56 ± 1.15^b^	2.00 ± 1.00^ab^	23.66 ± 1.52^b^	0.00 ± 0.00
**Preferencia social y tabla de pruebas de reflejos luminosos**					
**Ratas preñadas**					
**Grupos**	**FPS (%)**		**UPS (%)**		**LR (%)**
C	7.00 ± 2.82c		65.10 ± 3.53a		16.15 ± 0.25c
R	81.66 ± 2.35a		7.95 ± 1.90b		33.15 ± 0.16b
B	13.65 ± 0.49b		73.90 ± 3.74a		50.01 ± 0.40a
Y	12.30 ± 2.40bc		72.65 ± 3.74a		16.15 ± 0.25c
**Crías de ratas**					
C	2.03 ± 0.64^c^		88,32 ± 4.00^a^		33.17 ± 0.16^b^
R	58.14 ± 2.24^a^		27.22 ± 3.47^d^		62.25 ± 3.81^a^
B	18.69 ± 5.25^b^		59.81 ± 5.56^c^		16.25 ± 0.35^c^
Y	1.84 ± 0.32^c^		78.14 ± 2.50^b^		16.32 ± 0.33^c^
**Tabla de preferencia de la descendencia de la madre**			**OOPS (%)**		**UOPS (%)**	
C			30.52 ± 3.10b		56.11 ± 5.50a	
R			64.16 ± 1.01a		31.66 ± 2.35c	
B			29.16 ± 0.39b		42.31 ± 1.43b	
Y			30.27 ± 0.38b		53.30 ± 3.93a	

*Las diferencias entre las medias indicadas con la misma letra no son significativas al nivel p < 0,05.

*: C; Control, R; Rojo, B; Azul, Y; Amarillo, TSC; Tiempo pasado en el centro, TSP; Tiempo pasado en la periferia, NEEC; Número de entradas y salidas del centro, NSC; Número de cuadrados cruzados, ND; Número de defecaciones, FPS; Puntuación de preferencia por animales familiares, UPS; Puntuación de preferencia por animales no familiares, LR; Reflejo de luz, OOPS; Puntuación de preferencia por crías propias, UOPS; Puntuación de preferencia por crías no familiares.

El tiempo de permanencia en el centro (TSC) de las ratas gestantes fue de 18,50 ± 3,53 segundos (s) para el grupo R, de 5,50 ± 2,12 s para el grupo B, de 2,50 ± 2,12 s para el grupo C y de 1,50 ± 0,70 s para el grupo Y. El TSP fue de 298,50 ± 0,70 s para el grupo Y, de 296,50 ± 0,71 s para el grupo C, de 294,50 ± 2,12 s para el grupo B y de 277,50 ± 3,53 s para el grupo R. Del mismo modo, para las crías, el TSC fue de 43,33 ± 5,13 seg para el grupo R, 25,33 ± 4,04 seg para el grupo B, 22,66 ± 1,15 seg para el grupo Y, y 13,66 ± 5,13 seg para el grupo C. Los valores de TSP para las crías se midieron en 285,33 ± 3,51 seg para el grupo C, 276,56 ± 1,15 seg para el grupo Y, 274,66 ± 4,04 seg para el grupo B, y 256,66 ± 5,13 seg para el grupo R.

En las ratas gestantes, el NEEC fue el más alto en el grupo B con 4,33 ± 0,57 y el más bajo en el grupo Y con 1,66 ± 1,15. El NSC fue de 57,33 ± 2,30 en el grupo R, de 44,66 ± 6,35 en el grupo Y, de 37,66 ± 2,88 en el grupo B y de 13,33 ± 2,30 en el grupo C. En las crías, este valor fue el más alto para el NEEC en el grupo B, mientras que el NSC más alto fue en el grupo R. Los valores obtenidos en las crías fueron similares a los de las ratas gestantes.

En el estudio se observaron diferencias significativas entre los índices de FPS y UPS en ratas gestantes al nivel p < 0,05 (Tabla 2). El FPS más elevado se registró en 81,66 ± 2,35% en el grupo R, mientras que el valor más bajo correspondió al grupo C, con 7,00 ± 2,82%. El SAI fue de 73,90 ± 3,74% en el grupo B, 72,65 ± 3,74% en el grupo Y, 65,10 ± 3,53% en el grupo C y 7,95 ± 1,90% en el grupo R, respectivamente.

En el estudio se investigaron las preferencias de las crías de rata por sus madres y adultos desconocidos. Se determinaron diferencias significativas entre los grupos (p < 0,05). Las crías de las ratas expuestas a la luz roja preferían pasar más tiempo con sus madres, con una preferencia del 58,14 ± 2,24%. Por el contrario, las crías de otros grupos de color tendieron a favorecer a los individuos desconocidos. Las preferencias por la RL entre los grupos de estudio fueron significativamente diferentes al nivel p < 0,05. En las ratas preñadas, la mayor preferencia por la luz correspondió al grupo azul, con un 50,01 ± 0,40%, mientras que la menor preferencia se observó en los grupos C e Y, con un 16,15 ± 0,25%. En cuanto a las crías, el mayor valor de preferencia se calculó en el grupo R, con un 62,25 ± 3,81%, y el menor en el grupo B, con un 16,25 ± 0,35% (Tabla 2).

En el estudio se investigaron los efectos de la luz aplicada a las ratas preñadas sobre la preferencia de las crías tras el nacimiento (Tabla 2). Se determinaron diferencias significativas entre los grupos (p < 0,05). La mayor tasa de OOPS se registró en el grupo R, con un 64,16 ± 1,01%, mientras que la mayor UOPS correspondió al grupo control, con un 56,11 ± 5,50%. También se observaron diferencias significativas en los criterios de comportamiento entre los grupos (p < 0,05) (Figura 2-b). Los mayores niveles de confort en las evaluaciones de comportamiento social y de alimentación se identificaron en R, mientras que el mayor comportamiento de reposo se observó en C.

En el estudio, las observaciones realizadas a lo largo del día revelaron que las conductas más frecuentes observadas fueron las de amamantamiento con manta, pasivo y arqueado, en el orden indicado. También se observaron diferencias en las conductas de amamantamiento relacionadas con la longitud de onda de la luz, observándose el mayor amamantamiento con manta bajo luz roja (Figura 2-c).

## DISCUSIÓN

La luz ambiental y el color de la luz se encuentran entre los factores ambientales significativos que afectan directamente al embarazo, la maternidad y la infancia^(17)^. En el estudio, el mayor consumo de pienso y agua, así como la mayor ganancia de peso, se observaron en el grupo R. El menor consumo de pienso se observó en el grupo B, mientras que el menor consumo de agua y ganancia de peso se dieron en el grupo C. Se cree que las diferencias observadas en el consumo de pienso y agua y en la ganancia diaria de peso están influidas por las longitudes de onda de la luz aplicada. La luz roja adicional aplicada durante la gestación aumentó el consumo de pienso y agua, lo que se tradujo en un aumento de peso. Wren-Dail et al.^(18)^ informaron en 2016 de que las ratas macho Sprague-Dawley de 4–5 semanas de edad tenían un mayor consumo de alimento y agua y un mayor aumento de peso cuando se exponían a la luz roja. Nuestros hallazgos concuerdan con este resultado. En muchos estudios, se ha indicado que la longitud de onda de la luz afecta a la motivación de los individuos para comer, aumenta el apetito y la velocidad de ingesta, y también influye en la elección de alimentos poco saludables^(19)^. En los seres humanos, la exposición excesiva a la luz afecta al peso corporal: la exposición matutina reduce el peso corporal y la exposición tardía lo aumenta^(20)^.

En el estudio, se determinó que el peso medio de las crías oscilaba entre 5,90 ± 0,01 g y 4,91 ± 0,14 g. El valor más alto se observó en el grupo expuesto a la luz azul, mientras que el más bajo se observó en el grupo expuesto a la luz roja. El número de crías fue similar en todos los grupos. Sobre la base de los resultados, se sugiere que la luz azul puede tener un efecto de aumento de peso en el desarrollo de las crías, mientras que la luz roja puede tener un efecto de disminución. Balcıoğlu y Özdamar^(21)^ informaron de que el peso al nacer de las crías de rata oscilaba entre 5,65 ± 0,11 g y 5,02 ± 0,13 g^(21)^. Los valores determinados en el presente estudio son similares a los informados por Balcıoğlu y Özdamar (2020).

El nivel más alto de glucosa en sangre entre los grupos se observó en el grupo C el día 9 del estudio, mientras que el más bajo fue en el grupo B el día 15. A lo largo del estudio, se observó una disminución continua de los niveles de glucosa en sangre en las ratas del grupo Y. Cheung et al.^(22)^ informaron de que la luz tenía un efecto sobre el metabolismo de la glucosa. La identificación de diferencias en los niveles de glucosa en sangre entre los grupos con diferentes aplicaciones de luz es coherente con esta información.

La exposición al estrés ambiental durante el embarazo puede afectar a la maduración de las crías en desarrollo, lo que puede conducir a cambios en el aprendizaje, los comportamientos y las emociones más adelante en la vida^(23)^. En la prueba de campo abierto, una disminución de la distancia recorrida y de las visitas al centro es un indicador de estrés^(24)^. El mayor número de defecaciones se observó en el grupo Y, mientras que en las ratas gestantes y las crías del grupo R no se observó ninguna defecación, que es un signo de estrés y miedo.

Los animales prefieren la socialización a estar solos y son más proclives a acoger a nuevos individuos en su entorno. Esto les lleva a favorecer a los compañeros desconocidos frente a los familiares^(25)^. En el estudio, se observó que la longitud de onda de la luz aplicada afectaba a las diferencias entre las proporciones de FPS y UPS de las ratas preñadas, lo que puede explicarse por el hecho de que la luz roja aumenta la FPS en las ratas preñadas, mientras que la luz azul aumenta la UPS, al contrario.

Los vínculos olfativos establecidos con la madre durante el desarrollo temprano son cruciales para un desarrollo social saludable. Las etapas posteriores de la vida se ven influidas por las relaciones positivas y beneficiosas que se forman durante este periodo. Se han realizado numerosos estudios para identificar los vínculos olfativos entre las madres y sus crías^(26)^. En nuestro estudio, las crías de las ratas expuestas a la luz roja preferían pasar más tiempo junto a sus madres en comparación con las demás.

En el estudio, la tasa más alta de OOPS se determinó en el grupo R, mientras que la tasa más alta de UOPS se observó en el grupo C, lo que sugiere que la exposición a la luz roja durante la gestación puede aumentar los sentimientos de apego materno hacia las crías. Se identificaron diferencias significativas en los criterios conductuales entre los grupos, quepueden deberse tanto a los efectos directos de la longitud de onda de la luz sobre las conductas como a sus efectos secundarios a través de las vías neurales^(27)^. Las evaluaciones más elevadas de los comportamientos de confort, social y de alimentación se determinaron en el grupo R, mientras que el comportamiento de reposo más elevado se observó en el grupo C. El estrés materno durante la gestación provoca una reducción de los comportamientos sociales. La observación del comportamiento social más elevado en el grupo R indica el efecto reductor del estrés de la luz roja, en consonancia con los hallazgos de Laviola et al.^(28)^. Del mismo modo, en un estudio realizado en primates, se informó de que la luz roja promovía el comportamiento activo en los primates, mientras que la luz azul provocaba una disminución de la actividad^(29)^. Además, los efectos conductuales de la luz en los seres humanos se observan a menudo como disfunción conductual, depresión y trastornos psicológicos^(30)^. En el estudio, los comportamientos observados con mayor frecuencia durante el día fueron el arqueamiento, la pasividad y el amamantamiento con manta.

## CONCLUSIÓN

Se llegó a la conclusión de que la luz roja adicional aplicada durante la gestación tenía un efecto de aumento de peso en las ratas gestantes, al tiempo que reducía el peso al nacer de las crías y también tenía un efecto diurético. La luz azul tuvo un efecto de aumento de peso en las crías, junto con sus efectos preventivos del edema. La luz amarilla provocó una disminución constante del metabolismo de la glucosa durante el embarazo. Además, la luz roja aumentó el confort y los comportamientos sociales y alimentarios de las ratas preñadas, redujo el estrés tanto de las ratas preñadas como de las crías, aumentó la preferencia por individuos conocidos, incrementó los sentimientos de maternidad y apego hacia las crías, y afectó positivamente a la capacidad de distinguir el olor de la madre y a los sentimientos de apego hacia ella. Además, se espera que este estudio aporte ideas para las posteriores investigaciones que se realicen sobre estos efectos en otros mamíferos, como las ratas. Se necesitan más estudios experimentales para evaluar los efectos de la iluminación LED de diferentes colores sobre las características fisiológicas y conductuales de las ratas preñadas, las madres y los recién nacidos.

## DISPONIBILIDAD DE DATOS

Los datos estarán disponibles previa solicitud razonable.
